# Amygdala's involvement in facilitating associative learning-induced plasticity: a promiscuous role for the amygdala in memory acquisition

**DOI:** 10.3389/fnint.2012.00092

**Published:** 2012-10-10

**Authors:** Lily S. Chau, Roberto Galvez

**Affiliations:** ^1^Psychology Department, University of Illinois at Urbana-ChampaignChampaign, IL, USA; ^2^Beckman Institute, University of Illinois at Urbana-ChampaignChampaign, IL, USA; ^3^Neuroscience Program, University of Illinois at Urbana-ChampaignChampaign, IL, USA

**Keywords:** Pavlovian conditioning, eyeblink conditioning, fear conditioning, inhibitory avoidance, cerebellum, neocortex, thalamic reticular nucleus

## Abstract

It is widely accepted that the amygdala plays a critical role in acquisition and consolidation of fear-related memories. Some of the more widely employed behavioral paradigms that have assisted in solidifying the amygdala's role in fear-related memories are associative learning paradigms. With most associative learning tasks, a neutral conditioned stimulus (CS) is paired with a salient unconditioned stimulus (US) that elicits an unconditioned response (UR). After multiple CS-US pairings, the subject learns that the CS predicts the onset or delivery of the US, and thus elicits a learned conditioned response (CR). Most fear-related associative paradigms have suggested that an aspect of the fear association is stored in the amygdala; however, some fear-motivated associative paradigms suggest that the amygdala is not a site of storage, but rather facilitates consolidation in other brain regions. Based upon various learning theories, one of the most likely sites for storage of long-term memories is the neocortex. In support of these theories, findings from our laboratory, and others, have demonstrated that trace-conditioning, an associative paradigm where there is a separation in time between the CS and US, induces learning-specific neocortical plasticity. The following review will discuss the amygdala's involvement, either as a site of storage or facilitating storage in other brain regions such as the neocortex, in fear- and non-fear-motivated associative paradigms. In this review, we will discuss recent findings suggesting a broader role for the amygdala in increasing the saliency of behaviorally relevant information, thus facilitating acquisition for all forms of memory, both fear- and non-fear-related. This proposed promiscuous role of the amygdala in facilitating acquisition for all memories further suggests a potential role of the amygdala in general learning disabilities.

## Introduction

It is widely accepted that the more emotionally arousing an event is (whether positive or negative), the better the event will be remembered (Cahill and McGaugh, 1995; van Stegeren et al., 1998; Cruciani et al., 2011). Such emotionally arousing events have been shown to peripherally cause many physiological changes, such as increased cortisol levels and elevated dehydroepiandrosterone (Schwartz, 2002; Dickerson and Kemeny, 2004). Investigations of the neurobiology of emotion have similarly demonstrated that emotionally arousing events modulate glucocorticoid and epinephrine levels in the brain. Many of these investigations have further suggested that the amygdala plays a key role in regulating these biochemical changes by regulating our emotional response to an event. For example, brain imaging analyses in humans have demonstrated a positive correlation between the amount of amygdala activation and degree of emotional arousal (Cahill et al., 1996; Costafreda et al., 2008). Furthermore, patients with amygdala damage exhibit impairments in their ability to recognize and express emotion (Adolphs et al., 1994, 1995). These analyses, along with rodent and non-human primate studies of amygdala function (Thompson et al., 1977; Lukaszewska et al., 1980; Swartzwelder, 1981; Rosen and Davis, 1988) have suggested that the amygdala plays a central role in mediating our emotional response to an event.

In addition to regulating the response to an emotional event, further analyses have also demonstrated that amygdala activation is directly tied to how well the emotional event is remembered. For example, memory tests in humans have found a positive correlation between the level of consolidation and the extent of amygdala activation (Cahill et al., 1996; LaBar et al., 1998). Furthermore, amygdala lesions in various species, including humans (Cahill et al., 1995), have been shown to dramatically impair a subject's ability to remember an emotional event (Werka et al., 1978; Liang et al., 1982; Jellestad and Bakke, 1985; Peinado-Manzano, 1988). Likewise, pharmacological activation of the amygdala produces a dose-dependent enhancement of memory for emotionally-motivated behavioral paradigms (Liang et al., 1986, 1990; Introini-Collison et al., 1991, 1996). These, and other similar analyses, have strongly suggested that the amygdala plays a role in facilitating memory consolidation for emotionally arousing events.

Although most would agree with the amygdala's importance in memory consolidation, there is still debate regarding the amygdala's role as an actual site of memory storage versus simply modulating storage of memory in other brain regions. Many learning theories suggest that the most likely site for long-term memories is the neocortex (Eichenbaum et al., 1992; Squire et al., 2004). However, some findings suggest that an aspect of some memories is stored in the amygdala, especially with fear associative learning paradigms. The following review will discuss findings utilizing fear- and non-fear-motivated Pavlovian behavioral paradigms to illustrate our current understanding of how the amygdala facilitates memory acquisition and consolidation.

## Amygdala's role in memory storage

### Fear associative learning

Studies utilizing fear conditioning paradigms, a type of Pavlovian conditioning, have demonstrated that the amygdala plays a role in both acquisition and consolidation of cued-fear associative learning (Kim and Jung, 2006; Johansen et al., 2011). In this review, the term *subjects* will be used when similar findings have been reported with multiple species. In cued-fear associative learning, a subject learns to associate a cue, such as a light or tone, the conditioned stimulus (CS), with an unpleasant stimulus evoking fear, such as a footshock, the unconditioned stimulus (US). To measure the strength of the tone-footshock-association, subjects are presented with the same cue in a novel environment and the fear response is recorded. Support for the amygdala playing a key role in fear associative memories stems from a myriad of studies varying in techniques, including lesioning (Blanchard and Blanchard, 1972; Kapp et al., 1979; Iwata et al., 1986; Phillips and LeDoux, 1992), electrophysiological recordings (Applegate et al., 1982; Pascoe and Kapp, 1985) and pharmaceutical manipulations (Gallagher and Kapp, 1978; Gallagher et al., 1981). The following section will focus on findings illustrating the role of the amygdala in consolidating cued-fear associations.

#### Amygdala as a site of storage

Analyses of amygdala function with cued-fear-conditioning have led many to suggest that the amygdala acts as a possible site of storage for these associations. In support of this theory, studies have demonstrated that the amygdala plays an essential role in retrieval of long-term fear associations (Lee et al., 1996; Maren et al., 1996; Schafe et al., 2001; Gale et al., 2004). For example, findings demonstrated that rats with lesions to the basolateral amygdala 1-day, 2-weeks, 1-month (Lee et al., 1996; Maren et al., 1996) or 16-months (Gale et al., 2004) following cued-fear-conditioning exhibit significantly less freezing behavior compared to sham controls. Additionally, inactivation of the amygdala prior to retention testing results in significantly fewer conditioned responses (CRs), compared to controls (Muller et al., 1997). Furthermore, studies disrupting protein synthesis in the amygdala, a molecular mechanism believed to be important for long-term memory consolidation (Guzowski et al., 2000; Kandel, 2001), have demonstrated impairments in fear-related memory. For example, various studies have demonstrated that disruptions in protein synthesis in the amygdala following acquisition via infusion of a protein synthesis inhibitor impair fear memory retention (Schafe and LeDoux, 2000; Duvarci et al., 2008; Kwapis et al., 2011). These studies, collectively, provide strong support for the amygdala either playing an essential role in retrieval of fear memories or that the amygdala is a site of storage for long-term fear associations.

**Figure 1:**
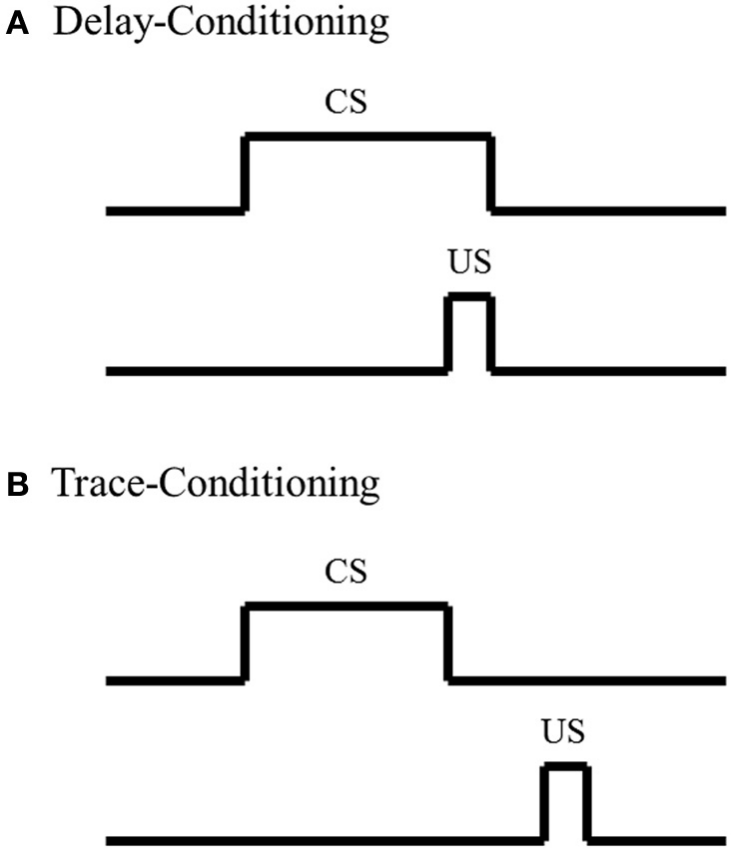
**Schematic of Pavlovian conditioning paradigms. (A)** In delay-conditioning, the conditioned stimulus (CS) (e.g., tone, whisker stimulation) co-terminates with the unconditioned stimulus (US) (e.g., mild footshock, eye shock). **(B)** In trace-conditioning, there is a stimulus-free separation in time between the CS and US.

To date, most investigations of amygdala's involvement in fear-conditioning, summarized in the discussion above, utilize a delay-conditioning paradigm; not many studies have examined the amygdala's role in a trace-fear-conditioning paradigm. In delay-conditioning, there is no separation in time between presentation of the CS and US. In contrast, there is a stimulus-free interval between the CS and US in trace-conditioning (Figure 1). Trace-fear-conditioning has been demonstrated to be dependent upon a number of distinct brain regions, including normal hippocampal (McEchron et al., 1998; Czerniawski et al., 2011) and medial prefrontal cortical activity (Runyan et al., 2004; Gilmartin and McEchron, 2005). However, the amygdala's role in trace-fear-conditioning is not as well understood as the hippocampus and medial prefrontal cortex. Raybuck and Lattal (2011) found that global amygdala inactivation via GABA_A_ agonist muscimol infusion prior to trace-fear-conditioning resulted in no significant differences in freezing behavior, compared to sham and vehicle controls, suggesting that acquisition for the trace-fear-association is independent of the amygdala. In contrast, studies have found that global amygdala inactivation via infusion of the same GABA_A_ agonist muscimol or blocking protein synthesis in the amygdala hinders acquisition for trace-fear-conditioning compared to controls (Kwapis et al., 2011; Gilmartin et al., 2012), suggesting that acquisition for the trace-fear association is dependent upon amygdala involvement. Although further analyses are needed to decipher the discrepancy between these findings, one possible explanation could reside in the extent of the amygdala inactivation. Studies have shown that different amygdala nuclei play specific roles in delay-fear-conditioning (Nader et al., 2001). Such nuclei specific analyses have not been as well examined with trace-fear-conditioning and could account for the conflicting findings. Although these analyses of amygdala function in trace-fear-conditioning conflict, analyses with delay-fear associations suggest that the amygdala is critically involved and could act as a possible site of storage for trace-fear associations.

#### Amygdala not as a site of storage

Although most analyses of cued-fear-conditioning suggest that the amygdala is a site of storage, most learning theories suggest that the neocortex is the most likely site of storage for long-term memories (Eichenbaum et al., 1992; Squire et al., 2004). In support of this theory, studies have demonstrated that training on an object orientation task, a paradigm where non-human primates learn to direct their attention toward a specific visual stimulus, alters both neuronal sensitivity and preferred orientation in primary visual neocortex (Schoups et al., 2001; Ghose and Maunsell, 2002). Likewise, rearing rodents in an enriched environment, a learning condition where subjects are reared in an environment facilitating enhanced motor, visual, and social stimulation, induces various forms of neocortical plasticity, such as increased dendritic material (Greenough and Volkmar, 1973; Juraska et al., 1980; Juraska, 1984) and increased number of dendritic spines in primary visual neocortex (Globus et al., 1973; Diamond et al., 1975; Turner and Greenough, 1985; Kolb et al., 2003). Furthermore, findings from frequency discrimination training, where a subject learns to preferentially favor a specific tone, have been shown to alter the preferred frequency receptive field in primary auditory neocortex (Disterhoft and Stuart, 1976; Kitzes et al., 1978; Kraus and Disterhoft, 1982; Diamond and Weinberger, 1986; Edeline et al., 1993; Recanzone et al., 1993; Rutkowski and Weinberger, 2005). Finally, studies utilizing tactile discrimination, where a subject learns to dissociate two tactile stimuli, have been shown to alter somatosensory neocortical map hand representation (Jenkins et al., 1990; Recanzone et al., 1992) and alter neuronal firing rate in primary somatosensory barrel neocortex (Krupa et al., 2004) for digit and whisker stimulation, respectively. These, and similar studies, along with various learning theories, have strongly suggested that the neocortex is modulated in response to learning and is a likely location for storage of most long-term memories.

In addition to these analyses suggesting that the neocortex is a likely site of long-term memory storage, some studies have also suggested that fear associations are not stored in the amygdala, but rather stored in other brain regions, such as the neocortex. These analyses have argued that the amygdala does not act as a site of consolidation for fear, but rather facilitates our ability to express fear. For example, studies have found that inactivation of the amygdala impairs freezing behavior in rodents when presented with cat fur, a non-learned stimulus that naturally induces fear in rodents (Vazdarjanova et al., 2001). These findings suggest that amygdala lesion-induced abnormalities in cued-fear-conditioning are due to an inability to express fear rather than removal of the site responsible for fear-related memory consolidation. Further support for this theory has come from analyses utilizing inhibitory avoidance conditioning. With inhibitory avoidance conditioning, a subject learns that a dark compartment CS is associated with an unpleasant stimulus, a footshock US. However, rather than demonstrating this learned association with a fear response, the rodent demonstrates the learned association by avoiding entering the dark compartment. Note, there are many variations of this paradigm that can add other forms of learning such as an operant component; however, for the purpose of this review, we will focus on the associative aspects. Studies utilizing the inhibitory avoidance conditioning paradigm have found that post-training amygdala lesions do not impair expression of the learned fear-association (Liang et al., 1982; Parent et al., 1995). These findings suggest that the amygdala is not a site of storage for inhibitory avoidance fear associations. Furthermore, these findings suggest that the amygdala may not be a site of storage for cued-fear-conditioning. However, the molecular analyses demonstrating that post-training amygdala infusion of protein synthesis inhibitors following cued-fear-conditioning impair memory retention (Kwapis et al., 2011; Gilmartin et al., 2012) disagree with these findings, and suggest that an aspect of the cued-fear memory is stored in the amygdala. Irrespective of the specific site of storage for fear associations, these, and other studies, have collectively demonstrated that the amygdala plays an essential role in either storing fear-related memories or facilitating consolidation of fear-related memories in other brain regions.

### Non-fear associative learning: eyeblink conditioning

The studies previously discussed, along with various others analyses examining amygdala function with fear-associative paradigms, have strongly suggested a role for the amygdala in fear associations; however, amygdala involvement in classic non-fear associative paradigms, such as eyeblink conditioning, are not as well understood. In eyeblink conditioning, a subject learns that a neutral stimulus CS, such as a tone or whisker stimulation, predicts delivery of a second stimulus US that elicits an eyeblink. After repeated CS-US pairings, the subject learns to blink when presented with the CS in anticipation of the US. In delay-eyeblink conditioning, the US co-terminates with the CS; thus there is no separation in time between the two stimuli (Figure 1). This form of learning is mediated by brainstem-cerebellar processing (Clark et al., 1984; Mauk and Thompson, 1987) and is not dependent upon neocortical processing (Norman et al., 1977; Oakley and Russell, 1977; Mauk and Thompson, 1987). Furthermore, various lesion and electrophysiological analyses have suggested that consolidation for delay-eyeblink associations occur in the cerebellum. For a detailed review of mechanisms for memory consolidations with delay-eyeblink-conditioning see Thompson and Steinmetz (2009). Based upon current understanding of the neuronal pathways necessary for delay-eyeblink-conditioning, the amygdala is not believed to play a prominent role in acquisition of the association (Thompson and Steinmetz, 2009). Furthermore, unlike fear associative paradigms, this form of conditioning is not predominantly believed to be fear-motivated. Although analyses of heart rate and blood pressure, factors that increase with fear, have demonstrated increased levels within the first few CS-US pairings, these properties decrease, while the associative behavior increases with conditioning (Hein, 1969; Powell and Kazis, 1976). These studies suggest that acquisition for eyeblink conditioning is not dependent upon fear, thus further suggesting that the amygdala would not play a dominating role in task acquisition. However, studies have found that under certain conditions, the amygdala does play a role in modulating acquisition for eyeblink associations.

#### Delay-eyeblink conditioning

**Figure 2:**
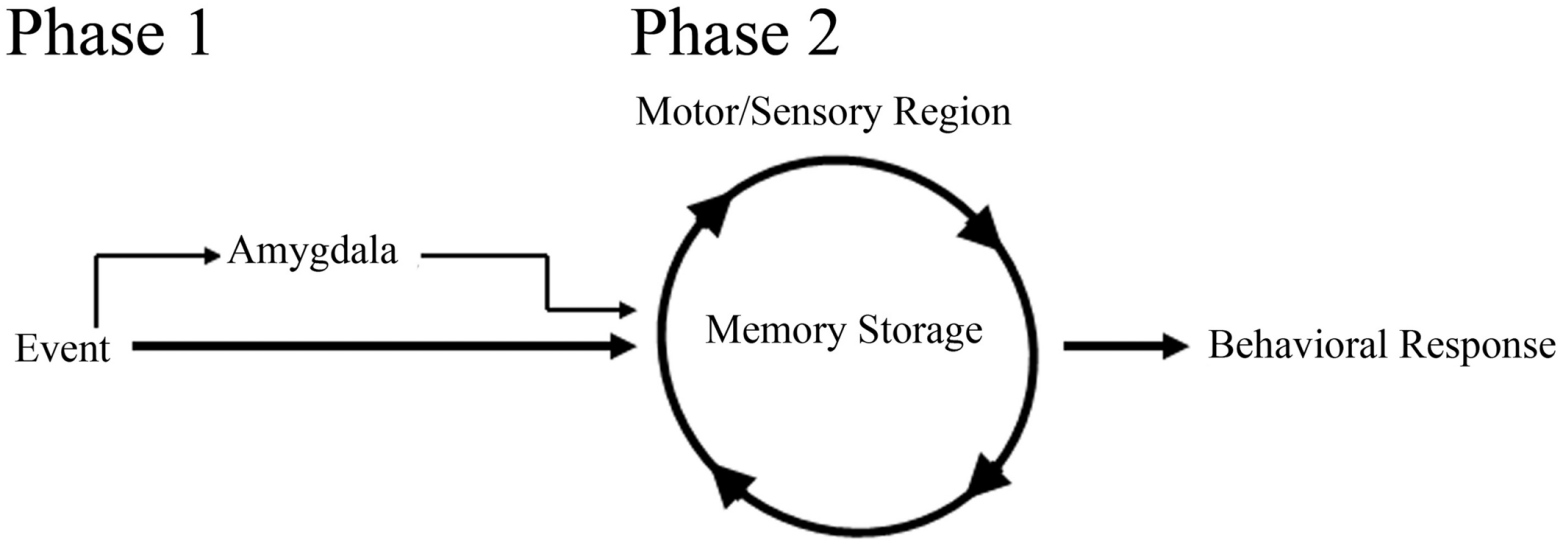
**Schematic of amygdala involvement in a two process model for memory consolidation.** In phase 1 of the model, the amygdala, receiving event information, increases the saliency of the event to motor and sensory regions, thus facilitating memory consolidation and behavioral response to the event. In phase 2 of the model, motor and sensory regions, primed with amygdala activation from phase 1, begin to solidify the memory and generate appropriate behavioral responses.

In support of a role for the amygdala in facilitating acquisition of eyeblink associations, studies examining delay-eyeblink-conditioning have found that amygdala stimulation increases the rate of acquisition for the association (Whalen and Kapp, 1991; Canli and Brown, 1996; Neufeld and Mintz, 2001). These studies strongly suggest that the amygdala can play a role in modulating memory for eyeblink conditioning, similar to fear associative learning paradigms. In support of this role, lesion studies have further suggested a more direct role for the amygdala in acquisition of eyeblink associations. Studies have found that post-training amygdala lesions do not have an effect on performance; however, pre-training amygdala lesions impair acquisition for the delay-eyeblink association (Weisz et al., 1992; Choi et al., 2001; Lee and Simons, 2004; Lindquist and Brown, 2004; Sakamoto and Endo, 2010). Furthermore, amygdala lesions have been found to reduce the rate of learning by dramatically impairing acquisition for the association during the initial days of training (Rescorla and Solomon, 1967; Choi et al., 2001; Mintz and Wang-Ninio, 2001; Lee and Simons, 2004). These findings suggest that the amygdala plays a critical role in enhancing the effectiveness of the CS early in training to assist with delivery of CRs. These, and other analyses of amygdala involvement in acquisition of the delay-eyeblink association, have offered support toward a two process model for consolidation (Figure 2). In this model, the initial phase of learning activates the amygdala and other emotional responses, possibly increasing the saliency of the CS. In the second (later) phase of learning, amygdala involvement decreases while motor and sensory regions solidify the association and generate well-timed CRs (Rescorla and Solomon, 1967; Choi et al., 2001; Mintz and Wang-Ninio, 2001; Lee and Simons, 2004). In support of this hypothesis, many non-specific emotional responses (e.g., increased heart rate and respiration) have been found to dissipate as appropriately timed CRs emerge (Hein, 1969; Powell and Kazis, 1976).

**Figure 3:**
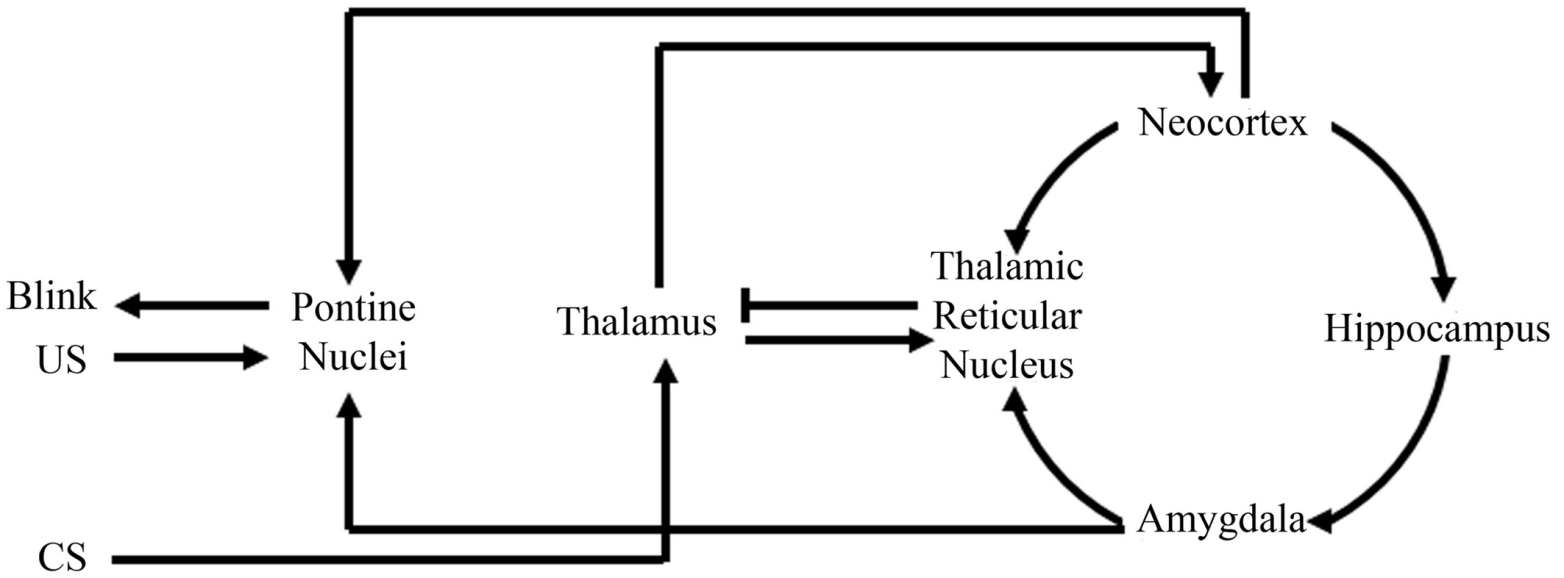
**Schematic of amygdala and thalamic reticular nucleus involvement with eyeblink conditioning.** Information from the conditioned stimuli (CS) first projects to the thalamus, where it will then project to the neocortex and thalamic reticular nucleus. The thalamic reticular nucleus can then compare information from the neocortex, amygdala, and thalamus. Then, via selective inhibition of thalamic activity, the thalamic reticular nucleus can modulate what information the neocortex receives. Modulation of neocortical input would modulate neocortical activation of the pontine nuclei that directly assists in generating the appropriate conditioned response “Blink.” Note in the above illustration, the amygdala can facilitate appropriate behavioral responses by not only modulating neocortical activation of the pontine nuclei via thalamic reticular nuclear stimulation, but also via direct projections to the pontine nuclei.

This theory, that the amygdala plays an initial role in learning by increasing the saliency of the behavioral events, is believed to be a general property in acquisition for other non-fear-motivated paradigms. Such a theory would suggest that the amygdala focuses one's attention on behaviorally relevant events or stimuli to facilitate acquisition and consolidation. In support of this argument, anatomical analyses of amygdala projections have found that the amygdala directly projects to the inhibitory thalamic reticular nucleus (TRN) (Zikopoulos and Barbas, 2012). The TRN receives projections from the neocortex and thalamus, but only sends inhibitory projections to the thalamus (Crick, 1984; Pinault, 2004), thus facilitating its ability to directly mediate or filter thalamocortical interactions (Figure 3). Further analyses have demonstrated that the TRN is activated when a subject is attending to a stimulus (Montero, 1997; McAlonan et al., 2008; Petrof and Brown, 2010). Furthermore, TRN lesions have been found to impair a rat's ability to attend to a stimulus (Weese et al., 1999). These findings, along with its anatomical connections facilitating inhibition of thalamic activation of the neocortex, have strongly suggested a role for the TRN in regulating what our brains are attending to (Crick, 1984; Pinault, 2004). Amygdala to TRN projections would allow the amygdala to directly modulate what information is conveyed to the neocortex. Such regulation would empower the amygdala to determine what our brains should attend to and thus would have tremendous implications toward more rapid acquisition of behaviorally relevant stimuli for any learning task (Figure 3).

Although the rodent literature has offered much support for the amygdala involvement in initial acquisition and this two process model for memory consolidation, not all studies examining amygdala involvement have supported this theory. Some rodent studies have observed a general reduction in the rate of acquisition with amygdala lesions (Sakamoto and Endo, 2010). Furthermore, studies using rabbits have suggested that the amygdala's involvement in delay-eyeblink-conditioning is not as prominent as suggested from rodent analyses. Analysis of delay-eyeblink-conditioning in rabbits have demonstrated only mildly impaired performance with amygdala lesions (Weisz et al., 1992). In their analysis, Weisz and colleagues (1992) further demonstrated that the impairing effects of amygdala lesions in rabbits can be diminished by increasing the intensity of the auditory stimulation used for the CS. These findings suggest that the saliency of the CS could have dramatic implications toward amygdala involvement and may account for possible discrepancies with amygdala lesions across species.

Another possible explanation for some of the discrepancies between these lesion studies could reside in the size of the lesion. Anatomically, it is known that the lateral amygdala receives converging input from both the auditory CS and somatosensory US pathways (Burton and Craig, 1979; LeDoux et al., 1987, 1990; Whalen and Kapp, 1991; Weisz et al., 1992). The lateral amygdala then projects to the basolateral amygdala and finally to the central amygdala. From the central amygdala, information projects directly to the pontine nuclei that then feeds information to the cerebellum. Although these regions are interconnected, there is no reason to believe each of these nuclei, or even every cell within each nuclei, would have equal involvement in acquisition for the delay-eyeblink association. Analyses of training-induced neuronal activation in the amygdala found that about 60% of the neurons responded to the CS while about 70% responded to the US (Richardson and Thompson, 1984). Thus, partial lesions could disproportionately alter the amygdala's involvement in delay-eyeblink associations. Furthermore, when neuronal activity from specific amygdala nuclei were examined, it was determined that unlike the central amygdala, which exhibited increased activity with conditioning, the basolateral amygdala did not exhibit a learning-specific pattern of activation (Rorick-Kehn and Steinmetz, 2005). Furthermore, additional analyses determined that although the central amygdala exhibited learning-specific activation, the extent of this activation could be modulated by simply varying the intensity of the US (Rorick-Kehn and Steinmetz, 2005). These findings strongly suggest that discrepancies in amygdala lesion studies could be due to differences in training conditions and the specificity of nuclei lesioned.

#### Trace-eyeblink conditioning

Although there are some inconsistencies in amygdala analyses, most studies suggest that the amygdala plays a critical role in acquisition of delay-eyeblink associations; however, analyses with trace-eyeblink-conditioning have not found that the amygdala plays as prominent of a role in acquisition of the association. In trace-eyeblink-conditioning, the CS and US are temporally separated by a stimulus-free interval (Figure 1). This form of learning is both hippocampal- and neocortal-dependent in that pre-conditioning lesions of the hippocampus and specific regions of the neocortex impairs a subject's ability to learn the trace-eyeblink association (Solomon et al., 1986; Moyer et al., 1990; Kim et al., 1995; McGlinchey-Berroth et al., 1997; Clark and Squire, 1998; Kronforst-Collins and Disterhoft, 1998; Weiss et al., 1999; Weible et al., 2000; McLaughlin et al., 2002; Takehara et al., 2002, 2003; Han et al., 2003; Tseng et al., 2004; Galvez et al., 2007). Unlike delay-eyeblink-conditioning, where consolidation for the association is believed to reside in the cerebellum, trace-eyeblink associations are believed to also reside in the neocortex. For example, analyses of neocortical plasticity following trace-eyeblink-conditioning have demonstrated unilateral learning-specific metabolic expansion of the primary neocortical area receiving input from the CS, compared to pseudo-conditioned controls (Galvez et al., 2006, 2011). Further analyses have demonstrated that neocortical lesions prevent acquisition for the trace-eyeblink association (Galvez et al., 2007). These, and other similar studies, have strongly suggested that the neocortex is a site of storage for trace-eyeblink associations.

With the neocortex acting as a site of storage for trace-eyeblink associations, most would speculate that the amygdala, similar to delay-eyeblink-conditioning, would play a role in facilitating consolidation. However, in trace-eyeblink-conditioning the amygdala does not appear to play as prominent of a role as observed in delay-eyeblink-conditioning. Analysis of metabolic activity in the central amygdala following eyeblink conditioning acquisition demonstrated increased activation with delay-eyeblink-conditioning; however, only a trend toward increased activation following trace-eyeblink-conditioning was observed (Plakke et al., 2009). Although this is only a single analysis, it suggests decreased involvement of the amygdala with trace-eyeblink-conditioning. However, based upon the two process model for consolidation (Figure 2) one would expect the amygdala to play a significant role during initial acquisition, but not once the association was learned. Furthermore, based upon the model, as the association is learned, the amygdala would decrease its involvement. This prediction of the model, along with the fact that trace-eyeblink associations require significantly more CS-US pairings, decreases the likelihood that the amygdala would still be activated following acquisition. Obviously, additional analyses of amygdala involvement in trace-eyeblink conditioning are necessary in order to make any definitive statements; however, analyses with delay-eyeblink-conditioning and the two process model for consolidation (Figure 2) suggest that the amygdala plays a role in facilitating initial acquisition for trace-eyeblink associations.

## Conclusion

Over the last several decades, there has been overwhelming evidence that the amygdala plays an essential role in facilitating acquisition and consolidation of fear associations. Although there is some question regarding the specific location of long-term memory storage (whether the amygdala or another region), these analyses strongly suggest that the amygdala plays a critical role in acquisition and consolidation of fear-related memories. However, the amygdala's role is not as clearly defined when examining non-fear-related memories. Utilizing eyeblink-conditioning as a non-fear-motivated task, this review suggests that there is also substantial support for amygdala involvement in acquisition of non-fear-motivated tasks. Analyses of amygdala involvement in these non-fear-motivated tasks suggest that the amygdala acts to increase the saliency of the learned stimuli so that other brain regions can consolidate the learned response. These findings suggest a two process model for memory consolidation. In this proposed model, the amygdala facilitates determining what thalamic information is conveyed to the neocortex. In support of this model, studies have found anatomical projections from the amygdala to the TRN, a brain region critically involved in directing attentional activation of the neocortex, the most likely site of storage for long-term memories. This model would suggest that amygdala lesions would decrease the rate of consolidation by not facilitating the initial phase of learning, but these lesions would not hinder a subject's ability to eventually acquire the association. These predictions are entirely consistent with the amygdala analyses with eyeblink conditioning mentioned above. Although this model was proposed under the framework of the eyeblink paradigm, the implications of these findings would have a broader role in other non-fear-motivated tasks. Additionally, such a model would also have a role in fear-motivated tasks. However, due to the amygdala's multifaceted role in different aspects of fear-motivated tasks, it is difficult to determine if the amygdala's role in modulating thalamocortical communication decreases during task acquisition similar to that of non-fear-motivated tasks. Together, these findings suggest that the amygdala plays a promiscuous role in directing our attention toward behaviorally relevant stimuli, thus facilitating acquisition and memory consolidation for both fear- and non-fear related memories. Currently, many analyses of the amygdala's role in humans have focused on individuals suffering from fear-related disorders such as post-traumatic-stress-disorder; however, the findings presented in this review demonstrate that the amygdala may also play a critical role in non-fear-related learning, suggesting that amygdala abnormalities could also plague many other neurological disorders of learning and memory.

### Conflict of interest statement

The authors declare that the research was conducted in the absence of any commercial or financial relationships that could be construed as a potential conflict of interest.
